# Factors Affecting Dengue Prevention Practices: Nationwide Survey of the Malaysian Public

**DOI:** 10.1371/journal.pone.0122890

**Published:** 2015-04-02

**Authors:** Li Ping Wong, Sharina Mahavera Mohamad Shakir, Narges Atefi, Sazaly AbuBakar

**Affiliations:** 1 Department of Social and Preventive Medicine, Faculty of Medicine, University of Malaya, Kuala Lumpur, Malaysia; 2 Julius Centre University of Malaya (JCUM), University of Malaya, Kuala Lumpur, Malaysia; 3 Department of Medical Microbiology, Faculty of Medicine, University of Malaya, Kuala Lumpur, Malaysia; 4 Tropical Infectious Diseases Research and Educational Centre (TIDREC), University of Malaya, Kuala Lumpur, Malaysia; Kaohsiung Chang Gung Memorial Hospital, TAIWAN

## Abstract

**Background:**

Efforts to stamp dengue in many dengue endemic countries has met little success. There is a need to re-examine and understand how the public at large view the dengue prevention efforts. This study aimed to examine the demographic factors, theoretical constructs of the Health Belief Model and knowledge about dengue and how these influence the practice of dengue prevention.

**Methods:**

A national telephone survey was carried out with 2,512 individuals of the Malaysian public aged 18–60 years.

**Results:**

The majority (73%) of the Malaysian public had a total dengue prevention score of 51–100 (of a possible score of 1–100). Multivariate analysis suggests significant correlates of higher dengue prevention practices with demographic background, perception of susceptibility to dengue, perceived density of mosquitoes in the neighbourhood and knowledge about dengue. Households of lower income of which the majority (40.7%) were from the rural areas, were associated with the highest odds [OR = 1.33; 95%CI = 1.09–1.67; p = 0.004] of dengue prevention. Dengue prevention practices were also less likely to be undertaken in neighbourhoods where the responders perceived there is no and/or low density of mosquitoes. Dengue prevention practices are also less likely to be practiced by skilled workers [OR = 0.78; 95%CI = 0.63–0.95; p = 0.029] compared to those unemployed. Higher perceived susceptibility to dengue was associated with higher dengue prevention practices and participants with higher dengue knowledge were found to have a higher level of involvement in dengue prevention practices.

**Conclusion:**

Results from the study suggest that in formulating approaches to contain dengue, strategies should be developed to cultivate dengue prevention practices among urban population and target areas with low density of mosquitoes where public perceived a less likely chance of getting dengue. Dengue prevention campaigns should focus on messages highlighting the risk of contracting dengue and education to increase knowledge about dengue.

## Introduction

Dengue is a mosquito-borne infection of particular health importance in Asia, the Americas and the Western Pacific. The infection has become endemic with frequent epidemic outbreaks [1] in many parts of the tropics and subtropical regions of the world. The World Health Organization (WHO) currently estimates there may be 50–100 million dengue infections worldwide every year including at least 500,000 dengue cases and 22,000 deaths, mostly among children. In Malaysia, dengue is endemic with frequent major outbreaks notably in urban areas [2]. Dengue is rated the most important communicable disease in Malaysia, superseding tuberculosis, malaria and HIV/AIDS [3]. The number of dengue cases reported was 21,900 in 2012 and 43,346 in 2013, representing an increase of over two fold in a one-year period [4]. As of August 2014, the number of suspected dengue cases has exceeded 60,000 proving to be the biggest outbreak yet of dengue in Malaysia.

Primary prevention which includes use of mosquito repellents, mosquito bed nets, mosquito coils, protective clothing and regularly removing sources of stagnant water to prevent mosquito breeding is suggested as the most effective measure in dengue prevention and control [2,5]. Ultra low volume fogging or conventional space spraying of chemical insecticides is carried out by the City Council in areas where there is a reported dengue outbreak. The success of efforts for prevention and control of dengue in the community, however, relies on the effectiveness of initiatives to educate the public about dengue and how it spreads, control of *Aedes spp* mosquito breeding sites by the general public and improving household environmental sanitation, water supply, and through sustained modification of human behaviour generally known as the Communication for Behavioural Impact (COMBI) [6]. It has long been recognized that socio-demographic characteristics, beliefs and practices about dengue have an impact on dengue prevention and control. Socio-demographic factors such as age, education and marital status influence the dengue prevention and control behaviour. In earlier studies, Al-Dubai et al. [7] found that those in the 31 to 40 year-old age group had higher dengue prevention practices in comparison to those aged 18 to 30 and to those aged ≥ 41 years. In addition, married couples reported higher dengue prevention practices compared to single people. These support the suggestion that individual’s health beliefs are likely to shape health care practices and often associated to health prevention behaviours. The Health Belief Model (HBM), one of the most widely used social cognition models to predict health behaviours, posits that individual's health behaviour is determined by four main elements: i) consideration of likelihood (susceptibility); ii) consideration of the seriousness (severity) of illness; iii) perceived benefits of taking health action; and iv) perceived barriers to taking health action. These four perceptions are elements that determine the readiness to take action and are activated by: i) cues to action and ii) self-efficacy [8]. The HBM has been used as a framework for understanding how to effectively structure health communication messages in order to change individual behaviour to prevent dengue [9,10].

Additionally, knowledge and awareness factors are also associated with prevention against dengue. Individuals with higher knowledge of dengue reported significantly higher practices of prevention measures than those with low knowledge about dengue [11,12]. Nevertheless, there is also contrasting evidence that implies that knowledge about dengue does not always result in the adoption of recommended preventive behaviours [13,14]. Past research conducted in Malaysia found that Malaysians generally have good knowledge of dengue and its prevention [5]. Nevertheless, little is known about the association between knowledge and prevention behaviours relevant to dengue.

Other factors that may influence people’s prevention practices contributing to the spread of dengue are living conditions such as house type and inhabitant density. Homes that are low-rise and clustered together are densely populated enabling easy mosquito transmission of infections between households. Researchers in Brazil showed that a high population density of low socioeconomic status, insufficient garbage collection and water supply provide ideal conditions for mosquito proliferation, especially *Aedes aegypti* [15]. In Thailand, villages near deciduous forest, horticulture and perennial areas strongly correlate with dengue indices. The density of vegetation is also a potential habitat for *Aedes spp* [16,17]. There is a higher chance of dengue outbreak occurring in areas where mosquito density is high. Mosquito density is a primary determinant for practicing mosquito avoidance measures. Papua New Guineans often sleep under a mosquito net when there are an abundance of mosquitoes during the rainy season [18]. In Africa, villagers use mosquito coils, spray and bed nets primarily because of annoyance from mosquito bites rather than the intent to prevent dengue or malaria [19].

The factors associated with dengue prevention practices and HBM, have not been fully explored in the Malaysian context where the infection has been exponentially increasing over the last few years. The current study, therefore, was undertaken to identify factors including demographic, theoretical constructs of the HBM and knowledge about dengue associated with the practice of dengue prevention. Identifying and understanding the influence of these factors on dengue prevention practices could facilitate the management of specific targeted factors in the overall prevention of dengue strategy.

## Methodology

### Sampling frame

Interviews were conducted between February 2012 and June 2013 using a computer-assisted telephone interview (CATI) system. The telephone numbers were generated randomly by the computer from the latest electronic residential telephone directory (2012/2013) of all 13 states in Malaysia. To be qualified for a telephone interview participants had to be Malaysians, between 18 to 70 years old, have heard of dengue and reside in the contacted household. Only one person per household was surveyed. If more than one qualified person was found in a household, one person was selected randomly using a random number table. Interviews were conducted between 5.30 pm and 10.00 pm on weekdays and from 12.00 pm to 7.00 pm on weekends or public holidays to avoid over-representation of unemployed participants. Unanswered calls were attempted at least two more times on separate days before being regarded as non-responses. Only one person per household was surveyed. If more than one eligible person is found in a household, one person will be selected in a separate random drawing from among all eligible participants.

### Instrument

The questionnaire consists of socio-demographic characteristics, experience of dengue, the HBM, knowledge of dengue and self-reported preventive practices against dengue.

1. Severity perception consists of two parts: perceived severity and perceived susceptibility to dengue, where perceived severity assesses feelings concerning the seriousness of dengue and perceived susceptibility assesses one's subjective perception of the risk of getting dengue.
2. Perceived severity was measured on a scale of 0–10 with a higher score indicating higher severity. Likewise, perceived susceptibility was measured on a scale of 0–10 with a higher score indicating higher susceptibility.
3. Perceived barrier examines perceptions of barriers to prevent dengue among participants. This was also measured on a scale of 0–10 with a higher score indicating greater barriers.
4. Self-efficacy: the opinion of being able to successfully manage dengue prevention behaviour. Self-efficacy was measured by a four-point Likert scale that ranged from 1 (strongly agree) to 4 (strongly disagree).

Knowledge related to dengue consist of seven parts, namely: (1) knowledge about dengue and the *Aedes spp*. mosquito, (2) knowledge about the transmission of dengue, (3) knowledge about prevention, (4) knowledge of signs and symptoms of dengue, (5) signs and symptoms of dengue hemorrhagic fever, and (6) knowledge about treatment, curability and precaution measure for people infected with dengue. The scale for the measurement of knowledge of dengue consisted of 44 items. For each statement, participants could choose between three response categories: 'yes', 'no' and 'don't know'. For the analyses, participants were scored as 1 for a correct response and 0 for an incorrect or 'don't know' response. Several negatively worded items were reversed and re-coded during the data analysis process. Possible scores ranged from 0 to 44. Higher scores indicate greater knowledge about dengue.

Practices regarding dengue prevention consist of three parts, namely: (1) prevention of mosquito breeding and (2) prevention of mosquito bites were assessed using nine-item and seven-item questions, respectively, and (3) prevention of dengue transmission was assessed using only one item. The options for practices, 'not at all', 'rarely', 'sometimes', 'often', and 'not applicable', were assigned a penalty point of 4, 3, 2, 1 and 0, respectively. Demographic questions (10 items) were asked after completion of the survey questions. To avoid problems inherent in translation, two bilingual experts translated the instruments from English to Bahasa Malaysia (National language of Malaysia), after which they were again blindly back-translated by two other bilingual experts. Content validity of the questionnaire was assessed by groups of experts to ensure that the items have acceptable content validity. The final draft version of the questionnaire was pilot tested.

### Ethical considerations

The study was approved by the Medical Ethics Committee, University Malaya Medical Center (UMMC), Kuala Lumpur, Malaysia (MEC 896.15). Participants were informed about the purpose and design of the study and assured that participation was voluntary and confidential. As written informed consent is not practical in a telephone survey, verbal informed consent was obtained from respondents prior to the beginning of an interview. The verbal consent method has been approved by the ethical review committee.

### Statistical analyses

All statistical analyses were performed with the Statistical Package for the Social Sciences version 20.0 (SPSS; Chicago, IL, USA). Non-responses and irrelevant answers were treated as missing values and therefore excluded from the analyses. Values of p ≤ 0.05 were considered significant. In addition to descriptive analyses, the chi-square test was used to test the significance of differences in percentages. Logistic linear regression analysis was conducted to determine factors associated with a higher dengue prevention practice score; these factors were socio-demographic (ethnicity, religion, occupation), density of mosquitoes in neighbourhood, ultra low volume (ULV) spraying of mosquito adulticides (fogging) frequency, the HBM and total knowledge score. Variables with a p-value <. 05 were entered into the logistic linear regression model.

## Results

### Participants’ characteristics

**Fig 1:**
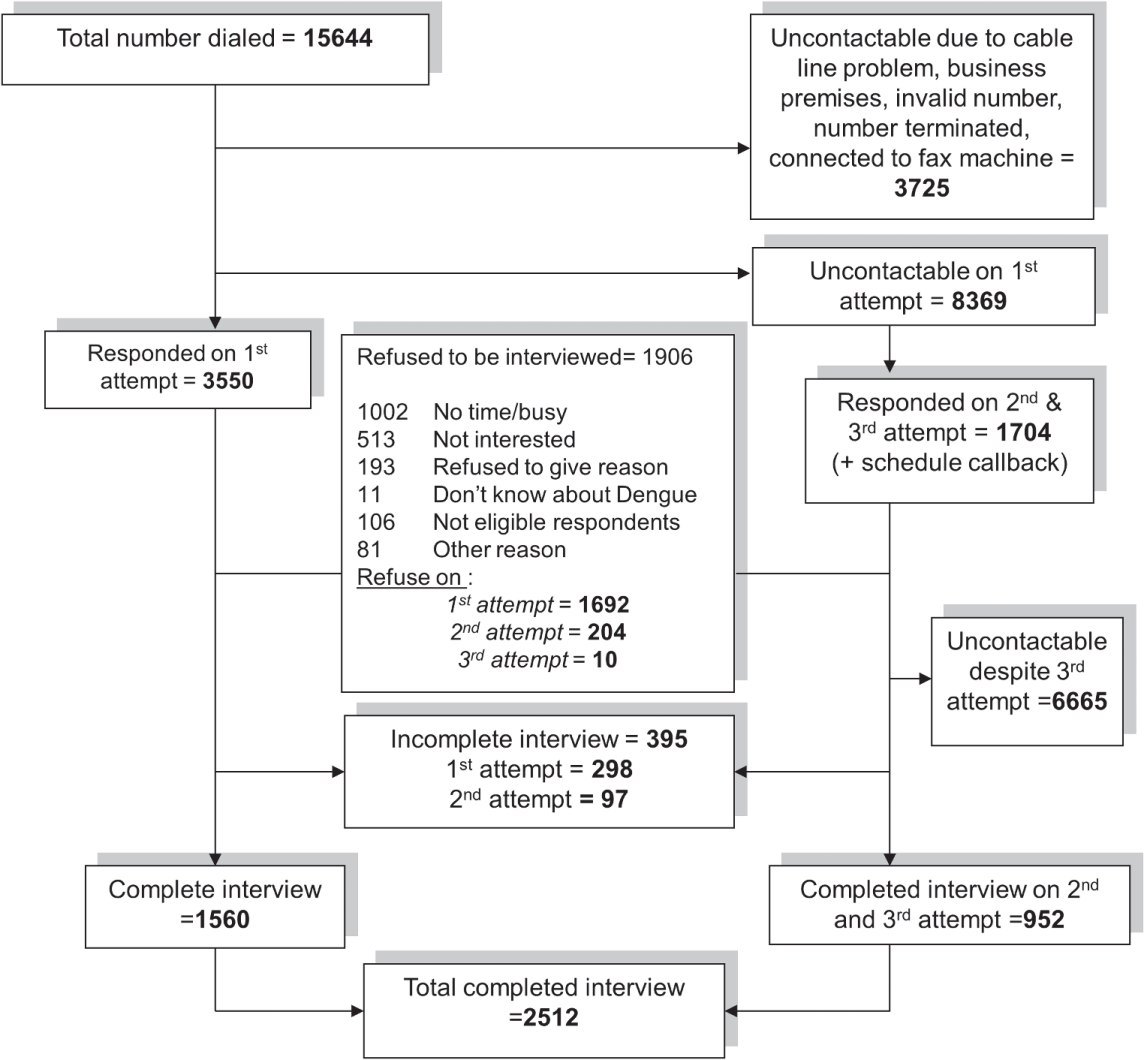
Illustration of the CATI process of the survey.

The flowchart of the CATI process is as shown in Fig 1. A total of 15,644 call attempts were made, resulting in 2,512 responding households. The response rate computed as the number of completed interviews (2,512) divided by the number of eligible and contactable households (5,354) was 46.0%. Participants who declined to participate in the survey were asked their reasons for not wanting to participate. The most common reasons for not wanting to participate were ‘busy’ (35.2%) and ‘not interested’ (18.0%). A total of 11 (0.3%) respondents said that they did not know about dengue and declined to be interviewed. Among the non-respondents, the majority were from the State of Selangor, one of the states with the largest urban populations.

The mean age of the study participants was 40.18 (±16.08) years, with an age range of 18 to 70 years old (Table 1). There were more females (64.3%) than males (28.5%) in this study. Most of the study participants had a secondary school education. The majority of participants were Malay (55.5%) with Chinese and Indians at 30.7% and 9.9%, respectively and the remaining consisted of and other ethnic minorities (3.9%). Less than half of the participants 31.5% (n = 790) had a monthly average income below MYR2000 (1USD = 3.19MYR). Participants with a higher income (more than MYR2000) [odd ratio (OR) = 1.88; 95% confidence interval (CI) = 1.58–2.24; p = 0.001 vs. income MYR2000 or less] were more likely to live in an urban area.

**Table 1:** Socio-demographic characteristics of respondents (N = 2512).

Socio-demographic variables	N	%
**Age**		
18–40	1272	50.6
>41 years	1240	49.4
**Gender**		
Male	717	28.5
Female	1795	64.3
**Marital Status**		
Single	766	35.7
Ever Married	1381	64.3
**Ethnicity**		
Malay	1394	55.5
Chinese	771	30.7
Indian	249	9.9
Others	98	3.9
**Religion**		
Muslim	1477	58.8
Christian	152	6.1
Buddhist	515	20.5
Others	268	14.7
**Highest educational Level**		
No formal education	43	1.7
Primary school	353	14.1
Secondary school	1292	51.4
Tertiary	824	32.8
**Occupation**		
Professional and managerial	333	13.3
Skilled worker	334	13.3
Non-skilled worker	396	15.8
Student	485	19.3
Housewife	702	27.9
Retired	205	8.2
Others	57	2.3
**Monthly average household income**		
Below MYR1000	303	12.1
MYR1001-MYR2000	487	19.4
MYR2001-MYR3000	411	16.4
MYR3001-MYR4000	233	9.3
MYR4001-MYR 5000	109	4.3
More than MYR5001	225	**9**
**Type of house**		
Flat/Apartment/ Condominium	468	18.6
Terrace house/twin house	1030	41
Bungalow/Village house	1041	40.4
**Does your house have a lot of plants or vegetation**		
None	147	5.9
Low	911	36.3
Moderate	676	26.9
A lot	778	31
**Your living area**		
Urban	864	34.4
Suburban	801	31.9
Rural	847	33.7

### Knowledge

**Fig 2:**
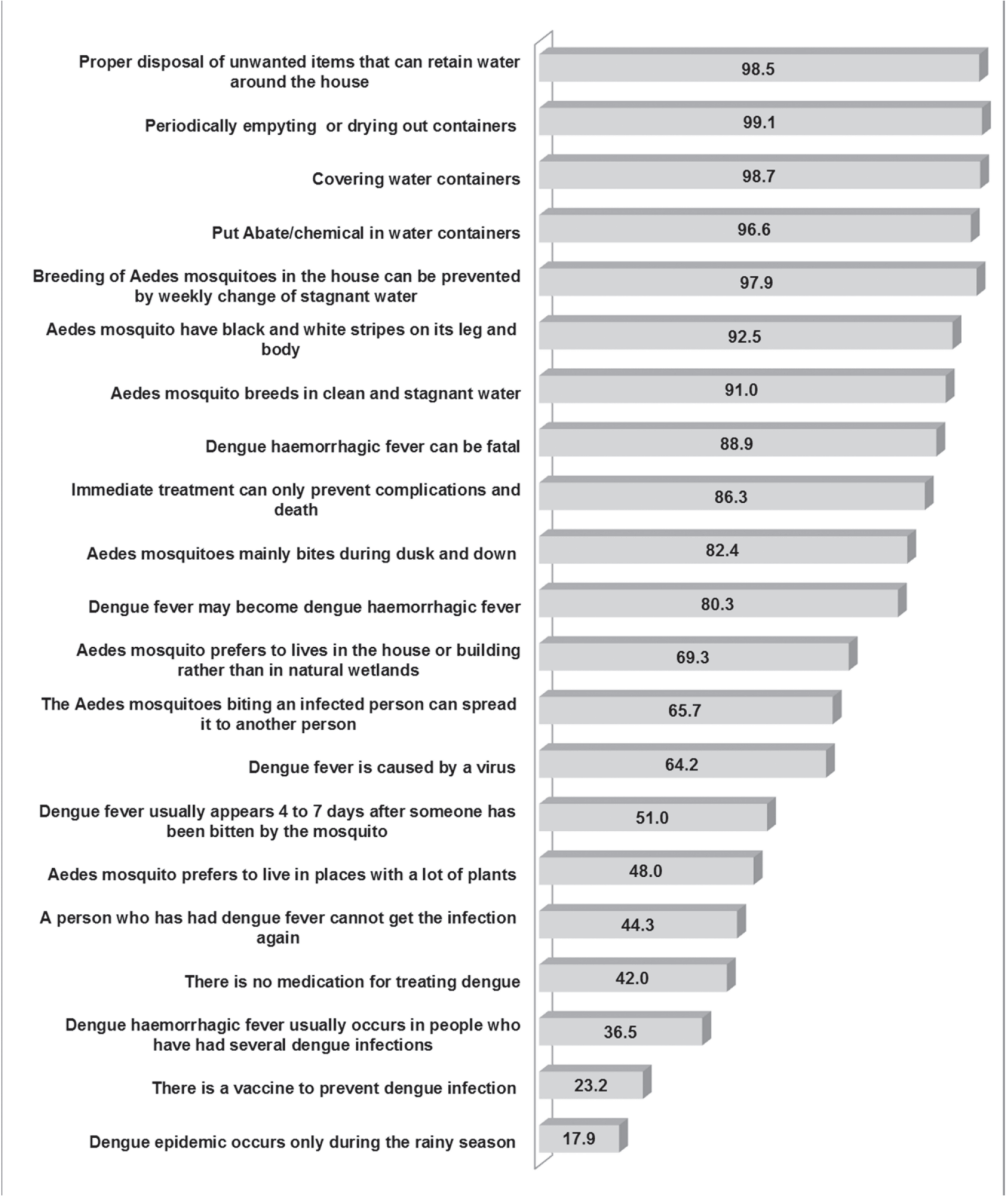
Percentage of knowledge related to dengue.

Approximately all of the participants (n = 2,465; 98.1%) were aware that mosquitoes transmit dengue and 91.8% (n = 2,308) of the participants knew that the dengue virus is transmitted specifically by the *Aedes spp*. mosquito (Fig 2). Most of the participants (n = 2,330; 92.8%) were aware that the *Aedes aegypti* mosquito has black and white stripes on its legs and body. More than one-third of the participants (n = 917; 36.5%) indicated that dengue haemorrhagic fever (DHF) usually occurs in people who had several dengue infections. Less than half of the participants (n = 1,205; 48.0%) reported that the *Aedes spp*. mosquito could live in places with a lot of plants. About half of the participants (n = 1,282; 51.0%) indicated that dengue usually appears four to seven days after someone has been bitten by a mosquito. Most of the participants (n = 2,261; 90.0%) were aware that fever is a symptom of dengue infections. However, only 39.2% (n = 985) of the participants knew that pain in the eyes is among the symptoms of dengue. Many of the participants (n = 1,930; 76.8%) were aware that there is no vaccine to prevent dengue infection. However, only about 42.0% of the participants (n = 1,055) indicated that there is no specific medication for treating dengue.

The mean total knowledge score for the overall sample was 27.49 (SD ± 8.34), out of a possible score of 42. As shown in Table 2, the total knowledge score was significantly different among gender, ethnicity, religion, educational level and occupation. There were a significantly higher proportion of participants with dengue experience that had a total knowledge score in the range of 23 to 44 compared to participants with no dengue experience. Higher perceived severity of dengue (level of severity 6–10) was found to have a higher proportion of total knowledge score in the range of 23 to 44 than a lower perceived severity of dengue (level of severity 0–5). The proportion of a total knowledge score range of 23 to 44 was significantly higher among participants with lower perceived susceptibility of dengue (level of susceptibility 0–5) than higher perceived susceptibility of dengue (level of susceptibility 6–10). Likewise, a significantly higher proportion of lower perceived barriers to prevent dengue (level of barriers 0–5) had a total knowledge score range of 23 to 44. It was also found that the proportion of a total knowledge score in the range of 23 to 44 was significantly higher for ‘disagreed’ on self-efficacy in the prevention of dengue than ‘agreed’ on self-efficacy in the prevention of dengue.

**Table 2:** Characteristic differences of participants, total knowledge score range, levels percentage of practice dengue prevention (%), and multivariate logistic regression analysis of variables associated to practice dengue prevention (N = 2512).

**Socio-demographic variables**	**Frequency**	**Knowledge score range**				**Practice Dengue Prevention by %**				**Logistic regression model 51–100 practice vs. 0–50 practice dengue prevention OR (95%CI)**
		0–22	23–44			0–50	51–100			
**Age**	**N(%)**	**N(%)**	**N(%)**	*P*		**N(%)**	**N(%)**	*P*		
18–30	925(36.8)	286(30.9)	639(69.1)	NS		250(27.0)	675(73)	NS		-
31–45	562(22.4)	175(31.1)	387(68.9)			157(27.9)	408(72.1)			-
More than 46	1025(40.8)	336(32.8)	689(67.2)			279(26.9)	749(73.1)			-
**Gender**										-
Male	717(28.5)	222(32)	495(68)	0.003		196(27.3)	521(72.7)	NS		-
Female	1795(64.3)	575(31)	1220(69)			487(27.1)	1308(72.9)			-
**Marital status**										
Single	766(35.7)	245(32)	521(68)	NS		207(27)	559(73)	NS		-
Ever Married	1381(64.3)	428(31)	953(69)			374(27.1)	1007(72.9)			-
**Ethnicity**										
Malay	1394(55.5)	360(25.8)	1034(74.2)	0.001		346(24.8)	1048(75.2)	0.001		1.18(0.67,2.07)
Chinese	771(30.7)	283(36.7)	488(63.3)			207(26.8)	564(73.2)			1.09(0.61,1.92)
Indian	249(9.9)	133(53.4)	116(46.6)			103(41.4)	146(58.6)			0.63(0.34,1.17)
Others	98(3.9)	21(21.5)	77(78.5)			27(27.6)	71(72.4)			Reference
**Religion**										
Muslim	1477(58.8)	387(26.2)	1090(73.8)	0.001		374(25.3)	1103(74.7)	0.001		0.76(0.42,1.38)
Christen	152(6.1)	49(32.2)	103(67.8)			36(23.7)	116(76.3)			1.28(0.78,2.09)
Buddhist	515(20.5)	193(37.5)	322(62.5)			142(27.6)	373(72.4)			1.0(0.65,1.52)
Others	368(14.6)	168(46.7)	200(54.3)			131(35.6)	237(64.4)			Reference
**Highest educational level**										
Primary school	396(15.8)	169(42.7)	227(57.3)	0.004		111(28)	285(72)	NS		-
Secondary school	1292(51.4)	402(31.1)	890(68.9)			342(26.5)	950(73.5)			-
Tertiary	824(32.8)	226(27.4)	598(72.6)			230(27.9)	594(72.1)			-
**Occupation**										
Skilled worker	667(26.6)	197(29.5)	470(70.5)	0.003		202(30.3)	465(69.7)	0.004		0.78(0.63,0.97)**
Non-skilled worker	396(15.8)	146(36.9)	250(63.1)			113(28.5)	283(71.5)			0.88(0.68,1.14)
unemployed	1449(57.7)	454(31.3)	995(68.7)			368(25.4)	1081(74.6)			References
**Monthly average household income**										
Below MYR2000	790(31.4)	244(30.9)	546(69.1)	NS		185(23.4)	605(76.6)	0.002		1.33(1.06,1.68)*
More than MYR2000	1722(68.6)	553(32.1)	1169(67.9)			498(28.9)	1224(71.1)			References
**Type of house**										
Flat/Apartment/ Condominium	468(18.6)	154(32.9)	314(67.1)	NS		126(26.9)	342(73.1)	NS		-
Terrace house/twin house	1030(41.0)	304(29.5)	726(70.5)			286(27.8)	744(72.2)			-
Bungalow/Village house	1041(40.4)	339(33.4)	675(66.6)			271(26.7)	743(73.7)			-
**Does your house have a lot of plants or vegetation**										
None	147(5.9)	45(30.6)	102(69.4)	NS		46(31.3)	101(68.7)	NS		-
Low	911(36.3)	264(29.0)	647(71.0)			258(28.3)	653(71.7)			-
Moderate	676(26.9)	221(32.7)	455(67.3)			183(27.1)	493(72.9)			-
A lot	778(31.0)	267(34.3)	511(65.7)			196(25.2)	582(74.8)			-
**Dengue experience**										
Yes(once)	148(5.9)	32(21.6)	116(78.4)	0.01		38(25.7)	110(74.3)	NS		-
No	2364(94.1)	765(32.4)	1599(67.6)			645(27.3)	1719(72.7)			-
**Density of mosquito in neighborhood**										
None	173(6.9)	62(35.8)	111(64.2)	0.001		62(35.8)	111(64.2)	0.001		0.56(036,0.87)*
Low	1334(53.1)	396(29.7)	938(70.3)			386(28.9)	948(71.1)			0.73(0.52,1.00)**
Moderate	730(29.1)	270(37)	460(63)			177(24.2)	553(75.8)			0.96(0.67,1.35)
Severe	275(10.9)	69(23.7)	206(74.6)			58(21.1)	217(78.9)			References
**Fogging frequency**										
None	398(15.8)	149(37.4)	249(62.6)	0.003		120(30.2)	278(69.8)			0.66(.044,1.07)
Rarely	1369(54.5)	459(33.5)	910(66.5)			395(28.9)	974(71.1)	0.004		0.72(0.50,1.04)
Occasionally	530(21.1)	149(28.1)	381(71.9)			126(23.8)	404(76.2)			0.88(0.56,1.26)
Often	215(8.6)	40(18.6)	175(81.4)			42(19.5)	173(80.5)			References
**Health Belief Model(HBM)**										
**Perceived Severity**										
0–5	201(8.0)	124(6.7)	77(38.3)	0.001		79(39.3)	122(60.7)	0.001		0.86(0.62,1.187)
6–10	2311(92.0)	673(26.1)	1638(70.1)			604(26.1)	1707(73.9)			Reference
**Perceived Susceptibility**										
0–5	1793(71.4)	550(30.07)	1243(69.3)	0.003		527(28.7)	1278(71.3)	0.006		0.76(0.61,0.94) **
6–10	719(28.6)	247(34.4)	472(65.6)			168(23.4)	551(76.6)			References
**Perceived Barriers**										
0–5	2002(79.7)	589(28.4)	1433(71.6)	0.001		527(26.3)	1475(73.3)	0.031		1.03(0.76,1.23)
6–10	510(20.3)	228(44.7)	282(55.3)			156(30.6)	354(69.4)			References
**Self-Efficiency**										
Agree	1747(69.5)	629(36)	1118(64)	0.001		474(27.1)	1273(72.9)	NS		-
Disagree	765(31.5)	168(22)	597(78)			209(27.3)	556(72.7)			-
**Knowledge Score**										
0–22	797(31.7)					318(39.9)	479(61.9)	0.001		0.42(0.34,0.51)*
23–44	1715(68.3)					365(29.1)	1350(70.9)			References

Hosmer and Lemeshow test, χ^2^ (8) = 15.86, p = 0.048; Cox and Snell R^2^ = 0.058; Nagelkerke R^2^ = 0.085

* association is significant at the 0.01 level

** association is significant at the 0.05 level

### Practice

The majority of participants (n = 1,892; 24.7%) reported that they cover water containers used for storing water in or outside the house and less than half of the participants (n = 1,178; 46.9%) reported that they put larvacide (Abate) or chemicals in water storage containers to prevent breeding of mosquitoes.

**Fig 3:**
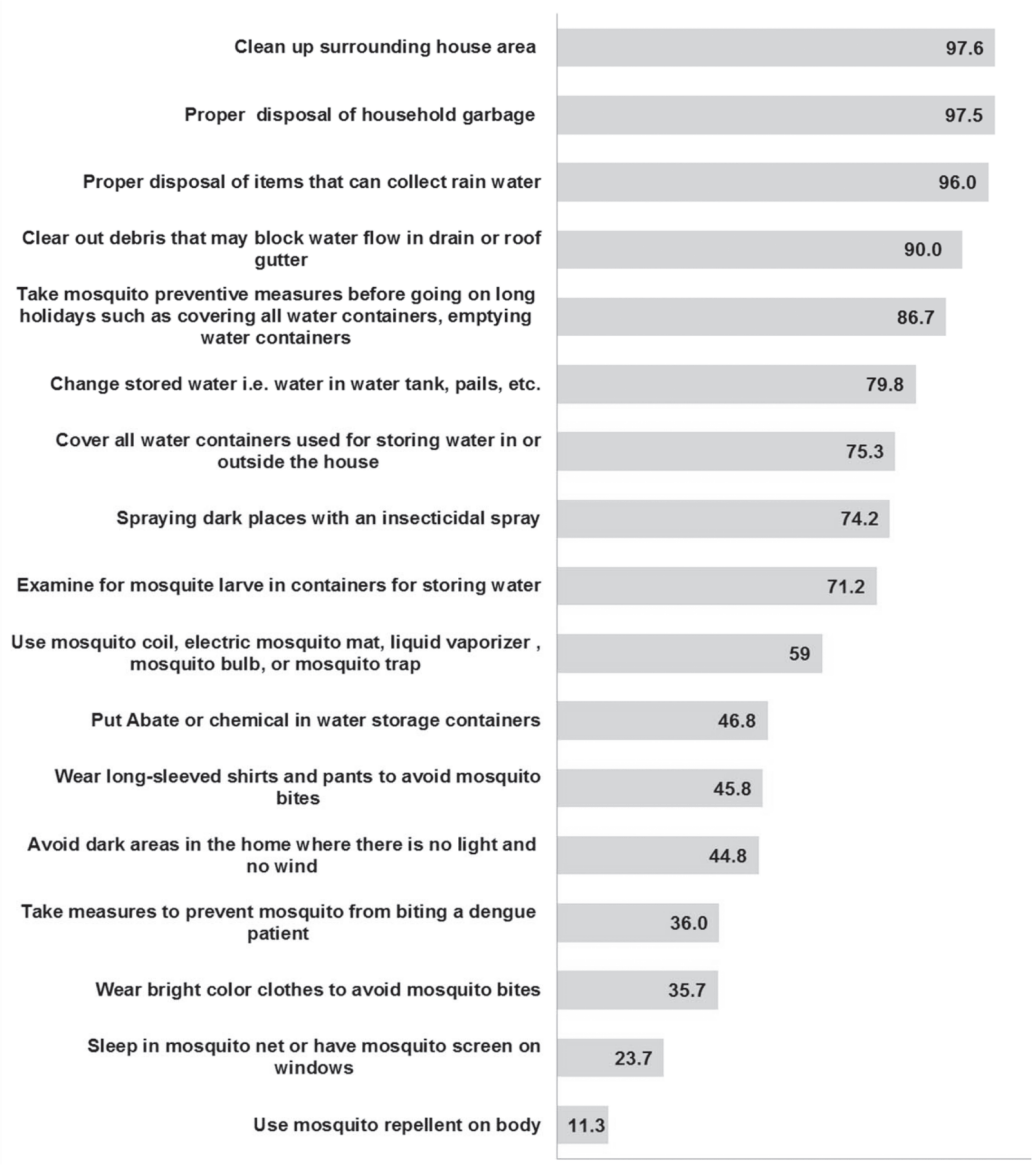
Percentage of practice dengue prevention.

Approximately all of the participants (n = 2,450; 97.5%) indicated that they clean up the surrounding house areas and 97.4% of the participants (n = 2,450) reported that they practice proper disposal of household garbage for dengue prevention. About one-fourth of participants (n = 597; 23.6%) indicated that they sleep in mosquito nets or have mosquito screens on windows. Only 11.3% of the participants (n = 283) reported that they use mosquito repellent on their body when outside the house. About one-third of participants (n = 894; 35.7%) reported that they wear bright coloured clothes to avoid mosquito bites (Fig 3).

In this study, the majority of the participants (n = 1,829; 72.8%) had a total dengue prevention practice score in the range of 51–100. The total dengue prevention practice score was significantly different among the socio-demographic variables, namely ethnicity, religion, occupation and monthly average household income. As shown in Table 2, a significantly higher proportion of participants that reported a severe density of mosquitoes in their neighbourhood had a total dengue prevention practice score in the range of 51–100. Higher perceived severity of dengue (level of severity 6–10) was found to have a higher proportion of a total dengue prevention practice score in the range of 51–100 than a lower perceived severity of dengue (level of severity 0–5). The proportion of a total dengue prevention practice score in the range of 51–100 was significantly higher among a higher perceived susceptibility of dengue (level of susceptibility 6–10) than a lower perceived susceptibility of dengue (level of susceptibility 0–5). A significantly higher proportion of lower perceived barriers to prevent dengue (level of barriers 0–5) had a total dengue prevention practice score in the range of 51–100 than higher perceived barriers to prevent dengue (level of barriers 6–10). It was also found that participants with a total knowledge score in the range of 23–44 had a significantly higher proportion of total dengue prevention practice score in the range of 51–100 than participants with a total knowledge score range of 0–22.

In Table 2, multiple logistic regressions indicated that the income group of less than MYR2,000 were more likely than the income group above MYR2000 to have higher dengue prevention practice score [OR = 1.33; 95% CI = 1.06–1.68; p = 0.013]. Skilled workers were less likely than the unemployed to have higher dengue prevention practice score [OR = 0.78; 95% CI = 0.63–0.95; p = 0.029]. Insignificant presence-(density) of mosquitoes in neighbourhood [OR = 0.56; 95% CI = 0.36–0.87; p = 0.001] was less likely to have higher dengue prevention practice score compared to the reference severe density of mosquitoes in neighbourhood. Lower perceived susceptibility (level of severity 0–5) has a lower likelihood [OR = 0.76; 95% CI = 0.61–0.94, p< 0.001] to have higher dengue prevention practice score compared to the reference level of severity 6–10. Participants with a lower total knowledge score range of 0–22 were less likely to have higher dengue prevention practice score [OR = 0.42; 95% CI = 0.34–0.51; p = 0.001] compared to the reference higher total knowledge score range of 23–44. Ethnicity, religion, ultra low volume (ULV) spraying of mosquito adulticides (fogging) frequency, perceived severity of dengue and perceived barriers to dengue prevention were not significant correlates of higher dengue prevention practice score.

## Discussion

Most of the participants of the study were aware that the *Aedes spp*. mosquitoes transmit dengue and a person with dengue may manifest symptoms including fever, joint and back pains, rashes and chills. However, only about one-third of the participants knew that dengue haemorrhagic fever usually occurs in people who had previous dengue infections. It was also found that less than half of the study participants were aware that there is no specific medication for the treatment of dengue. Therefore, it is recommended that dengue prevention educational programs should add focus on increasing the knowledge on symptoms of dengue including manifestation of severe dengue, unavailability of specific treatment for dengue and the importance of early detection of dengue.

The mean total knowledge score of 27 out of a possible score of 42 may reflect a moderate level of knowledge about dengue investigated in this study. Females showed a significantly higher knowledge score than males. Similarly in Puerto Rico, women are more concerned about the burden and health impact of dengue and household cleanliness related to elimination of mosquito breeding sites [20]. In Malaysia, it is a cultural norm that women play a greater role in the household and domestic activities, thus they play an active role in managing their family health. As dengue prevention should be a concerted effort of all members of the public regardless of gender, this implies that dengue prevention and control education should be more directed at men, emphasizing men taking responsibility for dengue prevention and encouraging them to be more aware and knowledgeable of dengue. Our study also suggests that respondents with tertiary education level had a significantly higher knowledge score. Previous research has shown that the level of education has a significant impact on knowledge related to dengue [21]. These findings suggest that knowledge of dengue is relatively low among people with lower education, thus more effective education programs for population awareness needs to be implemented among people with low education levels especially in large cities with high population density where dengue dominates.

Participants who had experience of dengue had a significantly higher knowledge score. This could perhaps be due to them actively seeking information when faced with the disease or having received information from healthcare staffs when undergoing treatment. These findings are consistent to that observed in Puerto Rico where those with a previous dengue diagnosis were more knowledgeable about dengue and concerned about others getting dengue [20]. Thus, knowledge about dengue and the importance of prevention should be stressed for people without dengue experience or in communities where dengue is not yet prevalent.

The dengue control program in Malaysia has focused on promoting household responsibility in eradicating mosquito breeding sites, specifically treating essential water-holding containers with larvicides, cleaning water containers weekly and house inspection for Aedes mosquitoes breeding [22]. This study found that in general households practiced good household cleaning practices in preventing mosquito breeding. Although the local authorities encourage use of the larvicide Abate, less than half of the respondents actually do so. Similar findings have been recorded in Pakse, a district in Laos with the highest number of dengue cases [23]. This may be due to the common perception that Abate is a harmful chemical. In addition the public may also have poor knowledge of how to use it and barriers to obtaining it [24]. Hence, besides distribution of the larvicides to neighbourhoods with dengue outbreaks, teaching the public to prevent mosquito breeding in water containers should include a demonstration on proper measurement and use of the larvicides. The public should be encouraged to add the larvicides in potential mosquito breeding sites and be informed of its safety properties.

The findings from the multivariate analysis suggest that significant correlates of dengue prevention practices were monthly average household income, occupation, perceived susceptibility to dengue, density of mosquitoes in the neighbourhood and knowledge of dengue, from the highest significant ORs to the lowest. Households with incomes below MYR2000 were associated with the highest odds of dengue prevention practice, of which the majority were in rural areas. The rural environmental surrounding that provides hospitable breeding grounds for mosquitoes and less busy lifestyle of people who live in rural or remote communities could be the reason of relatively higher dengue prevention practices among the people in rural areas. Therefore, dengue prevention and control campaigns should engage urban middle to higher income earners who mostly work away from home to allocate their time to carry out dengue prevention practices at home and in their neighbourhoods. Skilled workers were also less likely to practice dengue prevention compared to those unemployed. This could be because those who are unemployed, usually housewives, spend a lot of time at home and are most likely to carry out household cleaning activities. The multivariate findings from our study provide a base for creating educational and health messages for health behavioural change intervention based on the HBM constructs. It was found that higher perceived susceptibility to dengue was associated with higher dengue prevention practices. Thus, low perceptions of risk may reduce motivation to take action against dengue. Therefore, education programs need to highlight the equal risk of getting the disease to create awareness among people who are unaware of the serious threat of dengue. There should be more publicity in the mass media highlighting the high rate of infection among unsuspecting individuals especially among young adults, 21 to 40 years old where dengue cases are more prominent [25]. Previously, Pérez-Guerra et al. [20] suggested using testimonials from people with previous bout of dengue as a spokesperson for educational campaigns.

Our study also revealed that neighbourhoods with no significant presence and low density of mosquitoes were less likely to practice dengue prevention compared to neighbourhoods with severe density of mosquitoes. For this reason dengue prevention campaigns should also target neighbourhoods with a low prevalence of mosquitoes. Due to the less visible presence of mosquitoes, inhabitants may feel a false sense of security and neglect to take precautionary measures against dengue. Lastly, higher dengue knowledge translates into higher dengue prevention practice. This indicates that low knowledge of dengue transmission, prevention and treatment leads to poorer protective practices against dengue. Therefore, it is imperative to reinforce dengue education campaigns among people who have poor knowledge of dengue because this would encourage dengue prevention and control practices.

### Limitations of the study

Among the primary limitation of the study is that the computer-assisted telephone survey only included households with fixed line telephones; therefore, households without a telephone line (which are more likely to be from socio-economically disadvantaged groups) were under-represented [26]. The second limitation of this study is that all information obtained from the interview was self-reported and reporting bias due to socially desirable attitudes and behaviours might exist. Despite these limitations, the study presents findings of a nationwide study that features a relatively large sample size, which to our best knowledge is the first nationwide study in Malaysia.

## Conclusion

The study revealed that households with lower income, unemployed and unskilled workers carry out more dengue prevention practices. Therefore, households with middle to higher income earners, most of whom live in urban areas, and skilled workers should form the main targets of active dengue prevention and control campaigns. Secondly, from the HBM, higher perceived susceptibility of contracting dengue is associated with higher dengue prevention practices. As perceived susceptibility influences prevention practices, education should be specific about vulnerability to dengue. Thirdly, neighbourhoods with no significant presence or low density of mosquitoes were less likely to practice dengue prevention. Thus, these areas need to be integrated into dengue prevention programs to highlight the importance of mosquito prevention and control despite the perceived low mosquito problems. Lastly, higher dengue knowledge leads to higher dengue prevention practices. Therefore, knowledge-based education campaigns will substantially increase dengue preventive practices in the community. In summary, the study provides useful insights and knowledge that could guide the relevant authorities and government officials in planning, designing and initiating programs and activities aimed at preventing and control of dengue.

## Supporting Information

S1 Dataset (SAV)
